# Development of IL-15/IL-15Rα sushi domain-IgG4 Fc complexes in *Pichia pastoris* with potent activities and prolonged half-lives

**DOI:** 10.1186/s12934-021-01605-3

**Published:** 2021-06-09

**Authors:** Huan Xu, Mingyang Shi, Changsheng Shao, Hao Li, Jing Wu, Yin Yu, Fang Fang, Yugang Guo, Weihua Xiao

**Affiliations:** 1 Department of Oncology of the First Affiliated Hospital, Division of Life Sciences and Medicine, University of Science and Technology of China, Hefei, 230027 Anhui China; 2 Hefei National Laboratory for Physical Sciences at Microscale, the CAS Key Laboratory of Innate Immunity and Chronic Disease, School of Life Sciences, University of Science and Technology of China, Hefei, 230027 Anhui China; 3 Institute of Immunology, University of Science and Technology of China, Hefei, 230027 Anhui China; 4 Engineering Technology Research Center of Biotechnology Drugs Anhui, University of Science and Technology of China, Hefei, 230027 Anhui China

**Keywords:** Interleukin-15, Superagonists, Immunotherapy, Half-life, NK, *Pichia pastoris*

## Abstract

**Background:**

Interleukin-15 (IL-15) is a critical cytokine for the development, proliferation, and function of natural killer (NK) cells, NKT cells, and CD8^+^ memory T cells and has become one of the most promising protein molecules for the treatment of cancer and viral diseases. However, there are several limitations in applying IL-15 in therapy, such as its low yield in vitro, limited potency, and short half-life in vivo. To date, there are several recombinant IL-15 agonists based on configurational modifications that are being pursued in the treatment of cancer, such as ALT-803, which are mainly produced from mammalian cells.

**Results:**

In this study, we designed two different forms of the IL-15 complex, which were formed by the noncovalent assembly of IL-15 with dimeric or monomeric sushi domain of IL-15 receptor α (SuIL-15Rα)-IgG4 Fc fusion protein and designated IL-15/SuIL-15Rα-dFc and IL-15/SuIL-15Rα-mFc, respectively. The two IL-15 complexes were expressed in *Pichia pastoris* (*P. pastoris*), and their activities and half-lives were evaluated and compared. Pharmacokinetic analysis showed that IL-15/SuIL-15Rα-dFc had a half-life of 14.26 h while IL-15/SuIL-15Rα-mFc had a half-life of 9.16 h in mice, which were much longer than the 0.7-h half-life of commercial recombinant human IL-15 (rhIL-15). Treatment of mice with intravenous injection of the two IL-15 complexes resulted in significant increases in NK cells, NKT cells, and memory CD8^+^ T cells, which were not observed after rhIL-15 treatment. Treatment of human peripheral blood mononuclear cells (PBMCs) from healthy donors with the two IL-15 complexes yielded enhanced NK and CD8^+^ T cell activation and proliferation, which was comparable to the effect of rhIL-15.

**Conclusions:**

These findings indicate that the IL-15/SuIL-15Rα-dFc and IL-15/SuIL-15Rα-mFc produced in *P. pastoris* exhibit potent activities and prolonged half-lives and may serve as superagonists for immunotherapy in further research and applications.

**Supplementary Information:**

The online version contains supplementary material available at 10.1186/s12934-021-01605-3.

## Introduction

After decades of development, immunotherapy has become a promising strategy for cancer treatment [1]. Several recombinant cytokines for enhanced immune response have been developed as monotherapy agents or in combination with other therapies to improve therapeutic efficacy [2]. As one of the most promising immunotherapy drug candidates for cancer treatment [3], IL-15 was initially found to mediate immunostimulatory functions similar to IL-2, but it has unique properties. In addition to activating NK and cytotoxic T cells, IL-15 has the capacity to induce and maintain memory CD8^+^ T cells but not regulatory T cells and to inhibit IL-2-induced activation-induced cell death [4]. Thus, IL-15 has a great potential for clinical applications in the treatment of cancer.

Multiple preclinical studies have shown that IL-15 has potent antitumor activity [5]. A first-in-human clinical trial of rhIL-15 showed that IL-15 administration markedly increased the frequencies of NK cells, γδ T cells and CD8^+^ T cells in patients [6]. However, there are several limitations in the clinical application of IL-15, including its low yield in vitro, limited potency and short half-life in vivo. To improve the bioactivity of IL-15, a mutant, IL-15N72D, was generated to enhance the bioactivity by improving affinity of IL-15 to the human IL-15Rβ chain [7].

Physiologically, IL-15 transmits signals mainly through the trans-presentation mechanism, in which IL-15 is preassociated with IL-15Rα to form a heterodimer on the surface of monocytes and dendritic cells and then trans-presented to NK and CD8^+^ T cells expressing IL-15Rβ and γ chains [8]. With this information, several IL-15 superagonists consisting of IL-15 and partial (such as sushi domain, high-affinity IL-15 binding domain of human IL-15Rα) or whole IL-15Rα fused to the Fc domain of IgG1 have been designed and expressed in mammalian cells with enhanced immunostimulatory activity and extended half-lives [9–13]. These IL-15 superagonists exhibited an augmented antitumor effect compared to the IL-15 monomer in multiple studies, which pushed them (mostly ALT-803) into clinical trials to test their efficacy and safety in cancer treatment [13–16].

To date, more than ten Fc fusion proteins have been approved for marketing by the FDA and have achieved great commercial success [17]. Although a majority of therapeutic Fc fusion proteins are formatted on an IgG1 backbone, some side effects, such as target cell death and tissue damage caused by excessive cytokine secretion, are also introduced by the interaction of Fc with C1q or FcγRs [18]. In fact, for cases where the mechanism of action is mainly mediated by proteins fused to Fc, it may be desirable to have a silent Fc. One method is to employ other IgG subclasses, such as IgG2 or IgG4, which naturally have very limited ability to elicit effector functions compared with IgG1 [19]. In addition, multiple mutations in the Fc fragment could be introduced to further improve the safety and stability, including substitution in *N*-linked glycosylation sites and mutations that disrupt the interaction between Fc and C1q or FcγRs [18].

In this study, two different IL-15/SuIL-15Rα-IgG4 Fc complexes, including mutated IL-15 noncovalently bound to a modified dimeric SuIL-15Rα-IgG4 Fc (IL-15/SuIL-15Rα-dFc) and mutated IL-15 noncovalently bound to a modified monomeric SuIL-15Rα-IgG4 Fc (IL-15/SuIL-15Rα-mFc), were designed and expressed in *P. pastoris.* Nowadays, the methylotrophic yeast *P. pastoris* has been developed into a successful protein production platform. As a eukaryotic expression system, the increasing popularity of *P. pastoris* can be attributed to several factors, including its simple genetic manipulation, its cost-effective growth medium requirements, and its capacity to perform eukaryotic post-translational modifications, such as proper protein folding, proteolytic processing, disulfide bond formation and glycosylation [20, 21]. Moreover, *P. pastoris* can grow quickly to high cell densities and has high protein productivity, can produce up to grams amounts per liter of intracellular or secretory recombinant proteins [22, 23]. The rationale for designing IL-15/SuIL-15Rα-mFc with a smaller size and simpler structure is based on three considerations: (a) large proteins may have difficulties in extravasation and penetration into the tumor mass [24]; (b) the activity of IL-15 may be affected by the spatial conformation due to the potential steric hindrance between the two “arms” of Fc [25]; and (c) the binding rate of monomeric Fc to human FcRn is equivalent to that of normal Fc [26]. The biological activities and pharmacokinetic properties of the two complexes were evaluated in vitro and in vivo. The results indicate that the two IL-15/SuIL-15Rα-IgG4 Fc complexes have been successfully produced in *P. pastoris* with potent activities and prolonged half-lives and thus could be potentially applied in research and clinical practice.

## Results

### Molecular design and expression of IL-15/SuIL-15Rα-IgG4 Fc complexes

**Fig. 1:**
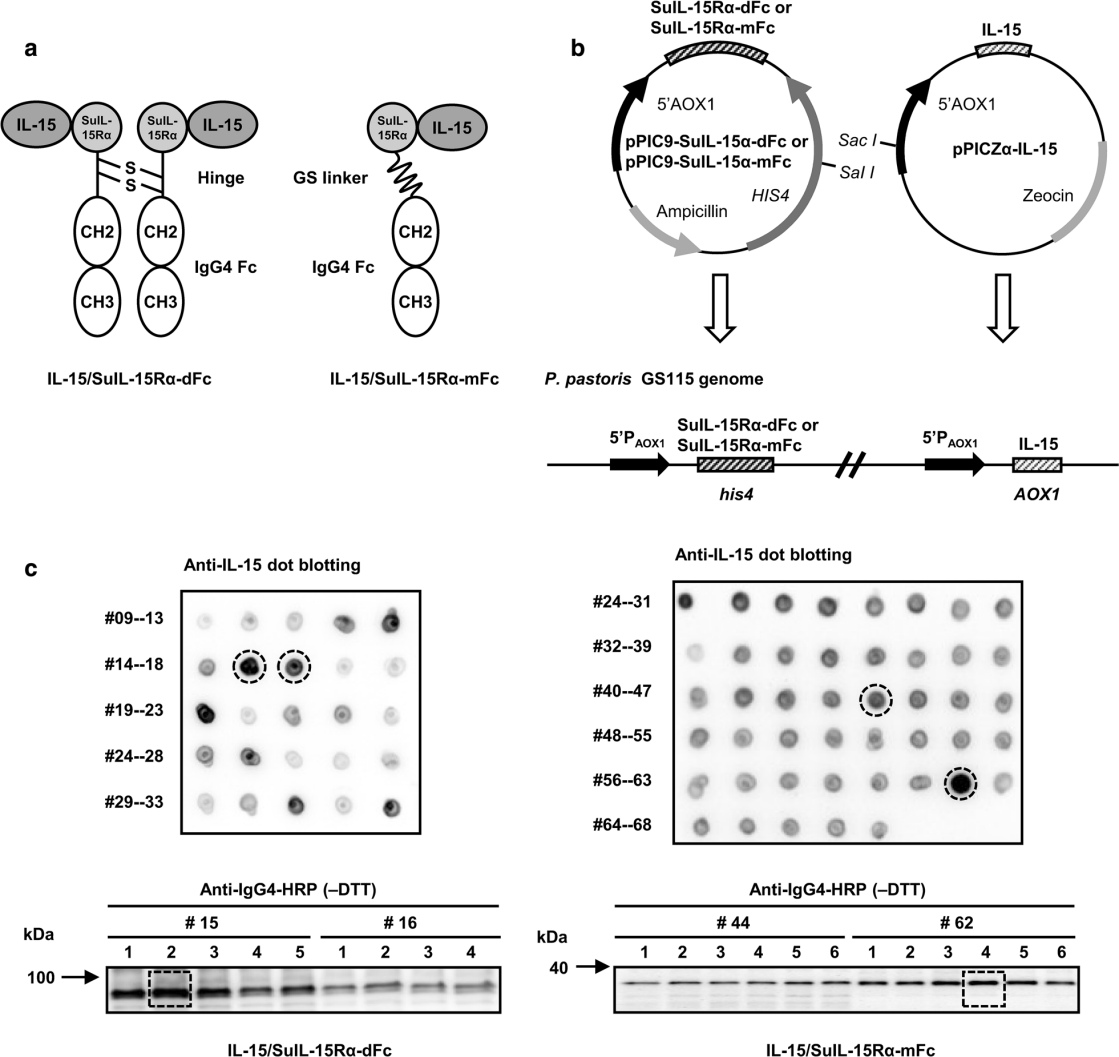
Construction and expression of IL-15/SuIL-15Rα-dFc and IL-15/SuIL-15Rα-mFc. **a** Schematic diagram of the two IL-15/SuIL-15Rα-IgG4 Fc complexes. IL-15/SuIL-15Rα-dFc was composed of two molecules of mutated IL-15 noncovalently bound to a modified dimeric SuIL-15Rα-IgG4 Fc fusion protein, while IL-15/SuIL-15Rα-mFc consisted of a single molecule of mutated IL-15 noncovalently bound to a modified monomeric SuIL-15Rα-IgG4 Fc fusion protein. **b** The construction strategy of the two IL-15/SuIL-15Rα-IgG4 Fc complexes. pPIC9-SuIL-15Rα-dFc or pPIC9-SuIL-15Rα-mFc and pPICZα-IL-15 were inserted into the genome of GS115 through homologous recombination using the *his4* and *AOX1* sequences, respectively. **c** Screening for the expression of IL-15/SuIL-15Rα-dFc and IL-15/SuIL-15Rα-mFc. Expression clones were first screened by dot blotting using rabbit anti-human IL-15 antibody followed by donkey anti-rabbit IgG-HRP conjugate, and then high expression clones were further selected and confirmed by Western blotting under nonreducing conditions using anti-human IgG4-HRP conjugate. Clones No. 15–2 and 62–4 were selected as the expression clones used in pilot-scale fermentation of IL-15/SuIL-15Rα-dFc and IL-15/SuIL-15Rα-mFc, respectively. The numbers in the figure represent the clone numbers

The IL-15/SuIL-15Rα-IgG4 Fc complexes composed of the human IL-15 moiety and SuIL-15Rα-IgG4 Fc fusion were generated in dimeric and monomeric formats, respectively. Previous studies have shown that the sushi domain (Su) of IL-15Rα (SuIL-15Rα), which locates in the N-terminal of SuIL-15Rα-dFc or SuIL-15Rα-mFc, bears most of the structural elements responsible for IL-15 binding [9, 11], and thus are able to form heterodimeric complexes with IL-15 in the endoplasmic reticulum of *P. pastoris*. As shown in Fig. 1a, IL-15/SuIL-15Rα-dFc contained a modified dimeric SuIL-15Rα-IgG4 Fc fusion protein which was linked by interchain disulfide bonds, and could bind two molecules of mutated IL-15. Compared with IL-15/SuIL-15Rα-dFc, IL-15/SuIL-15Rα-mFc became a monomeric format by replacing the full hinge region with a (Gly_4_Ser)_3_ linker. The mutated IL-15 protein was generated by introducing four amino acid substitution mutations in wild-type human IL-15: N71Q/N72D/N79Q/N112Q. The modified SuIL-15Rα-IgG4 Fc fusion protein was generated by introducing three mutations into the Fc fragment of IgG4: S228P/L235E/N297Q. The function corresponding to each mutation was listed in Table 1.

**Table 1:** Function of mutations in IL-15/SuIL-15Rα-IgG4 Fc complexes

Mutation	Function
IL-15	
N71Q	Remove *N*-glycosylation modification
N72D	Increase binding affinity to human IL-15Rβ, enhance biological activity of IL-15
N79Q	Remove *N*-glycosylation modification
N112Q	Remove *N*-glycosylation modification
IgG4 Fc	
L235E	Eliminate FcγR binding, reduce effector function
S228P	Prevent Fab-arm exchange, stabilize the disulfides in the core hinge
N297Q	Remove *N*-glycosylation modification

The coding sequence of SuIL-15Rα-dFc or SuIL-15Rα-mFc was inserted into pPIC9 vectors containing functional histidinol dehydrogenase gene (*HIS4*), while the coding sequence of mutated IL-15 was inserted into pPICZα vectors. These two vectors were inserted into the genome of the GS115 strain through homologous recombination (Fig. 1b). All three genes were fused with the α-factor signal sequence and under the control of the AOX1 promoter.

To obtain a high-expression strain for each IL-15/SuIL-15Rα-IgG4 Fc complex, the expression vector pPIC9-SuIL-15Rα-dFc or pPIC9-SuIL-15Rα-mFc was first transformed into *P. pastoris* strain GS115 via electroporation and then spread on histidine-deficient minimal dextrose (MD) plates. The high-expression clones of pPIC9-SuIL-15Rα-dFc and pPIC9-SuIL-15Rα-mFc were selected from positive transformants by Western blotting and then subjected to further transformation with pPICZα-IL-15. The transformants were plated onto YPD plates containing 1 mg/mL zeocin. The high-expression clones of the two IL-15/SuIL-15Rα-IgG4 Fc complexes were first screened by dot blotting, and then the selected clones were further confirmed by Western blotting. One of these high-expression clones (#15–2 for IL-15/SuIL-15Rα-dFc and #62–4 for IL-15/SuIL-15Rα-mFc) was used in the following pilot-scale fermentation (Fig. 1c).

### Fermentation, purification and characterization of IL-15/SuIL-15Rα-IgG4 Fc complexes

**Fig. 2:**
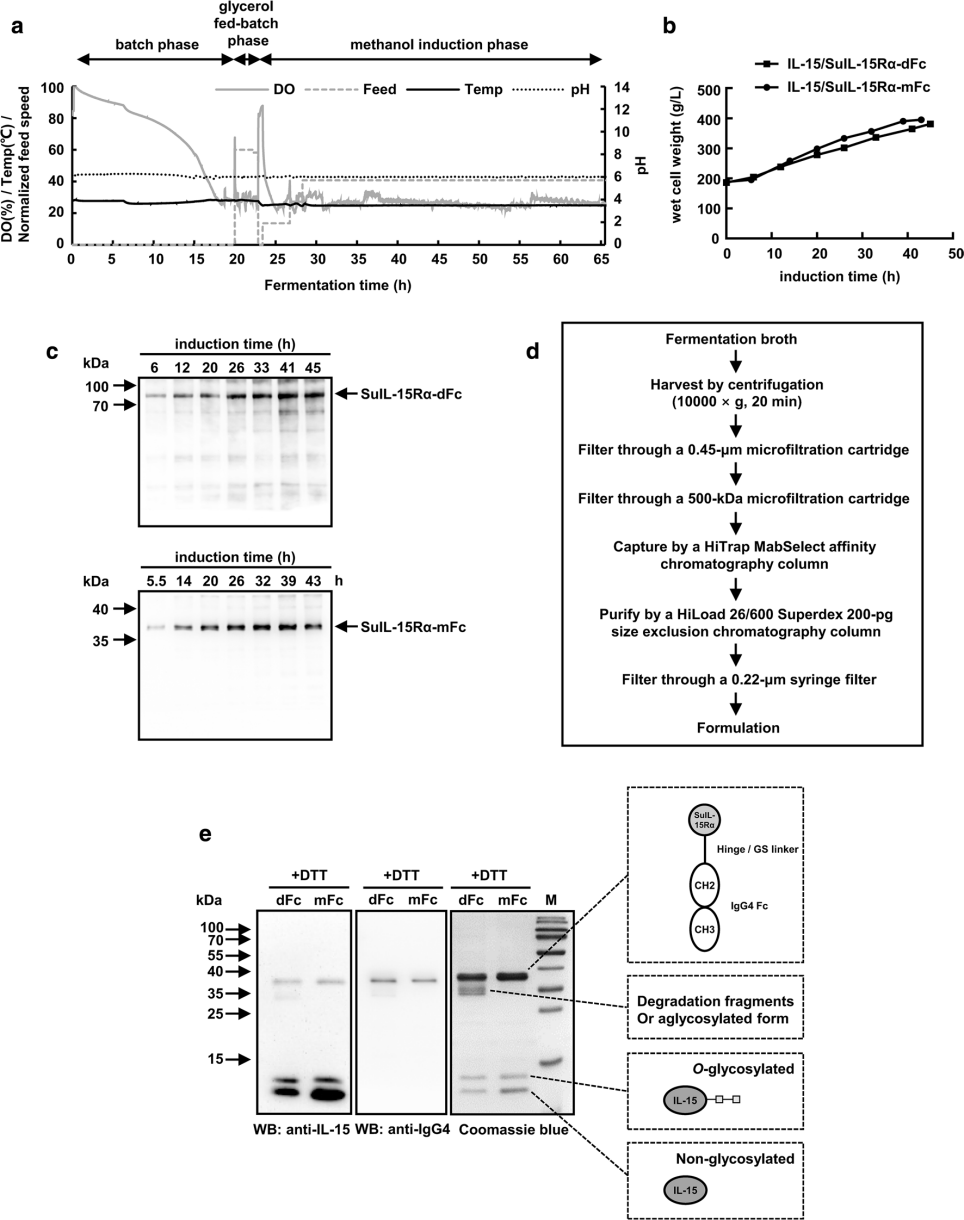
Pilot-scale fermentation, purification and characterization of IL-15/SuIL-15Rα-dFc and IL-15/SuIL-15Rα-mFc. **a** Representative three-step fermentation process, including batch phase, glycerol fed-batch phase and methanol induction phase. Parameters, such as dissolved oxygen (DO), feeding speed, temperature and pH, were monitored during fermentation. **b** Cell growth of the two IL-15/SuIL-15Rα-IgG4 Fc complex-expressing strains during fermentation was monitored and represented as wet cell weight. **c** The expression of the two IL-15/SuIL-15Rα-IgG4 Fc complexes during fermentation was analyzed by Western blotting using an anti-human IgG4-HRP conjugate under nonreducing conditions. **d** The downstream processing workflow of fermentation broth. **e** The two purified IL-15/SuIL-15Rα-IgG4 Fc complexes were separated by SDS-PAGE under reducing conditions and identified by Western blotting or Coomassie blue staining. The contents within the dashed boxes are the presumed protein structure of the corresponding bands. M: prestained protein marker; dFc: IL-15/SuIL-15Rα-dFc; mFc: IL-15/SuIL-15Rα-mFc

The typical three-step fermentation process included the batch phase, glycerol fed-batch phase, and methanol induction phase (Fig. 2a). The production of IL-15/SuIL-15Rα-dFc and IL-15/SuIL-15Rα-mFc gradually accumulated after methanol induction, and the yeast wet cell weight (WCW) reached 381 g/L and 395 g/L at the end of fermentation, respectively (Fig. 2b). The fermentation process lasted less than 45 h for IL-15/SuIL-15Rα-dFc or less than 43 h for IL-15/SuIL-15Rα-mFc (Fig. 2c) to avoid the degradation of the protein complexes. Furthermore, at the end of fermentation, the percentage of IL-15/SuIL-15Rα-dFc and IL-15/SuIL-15Rα-mFc in the fermentation supernatant was approximately 45% and 86% in all forms of recombinant IL-15 and SuIL-15Rα/Fc by densitometric measurement, respectively. After fermentation, the supernatant was collected by centrifugation and filtration, and then IL-15/SuIL-15Rα-IgG4 Fc complexes were captured by HiTrap MabSelect affinity chromatography and further purified by HiLoad 26/600 Superdex 200-pg size exclusion chromatography (Fig. 2d). The bacterial endotoxin levels of the final purified IL-15/SuIL-15Rα-IgG4 Fc complexes were below 0.01 EU/μg protein.

The purified protein complexes were examined by SDS-PAGE and Western blotting (Fig. 2e). The results of Western blotting confirmed the presence of both the IL-15 and Fc fragments of IgG4 in these complexes. Under reducing conditions, both IL-15/SuIL-15Rα-dFc and IL-15/SuIL-15Rα-mFc exhibited three bonds with MW values of ~ 38 kDa, 13 kDa and 12 kDa. These results indicate the disassociation of the IL-15 mutant and the SuIL-15Rα-Fc fusion because these molecular weights closely match the calculated MW of SuIL-15Rα-Fc (33 kDa) and mutant IL-15 (13 kDa), which is consistent with a previous study [11]. The 13-kDa band may be the modification form of the IL-15 mutant (e.g., *O*-glycosylation). Consistent with the previous report, there was a clear shift between *O*-glycosylated and non-glycosylated IL-15, which might indicate that IL-15 was modified by a chain of mannoses [27]. In addition, Coomassie brilliant blue staining revealed that some degradation fragments or aglycosylated form may exist in the protein sample of IL-15/SuIL-15Rα-dFc.

### In vitro bioactivity of IL-15/SuIL-15Rα-IgG4 Fc complexes

**Fig. 3:**
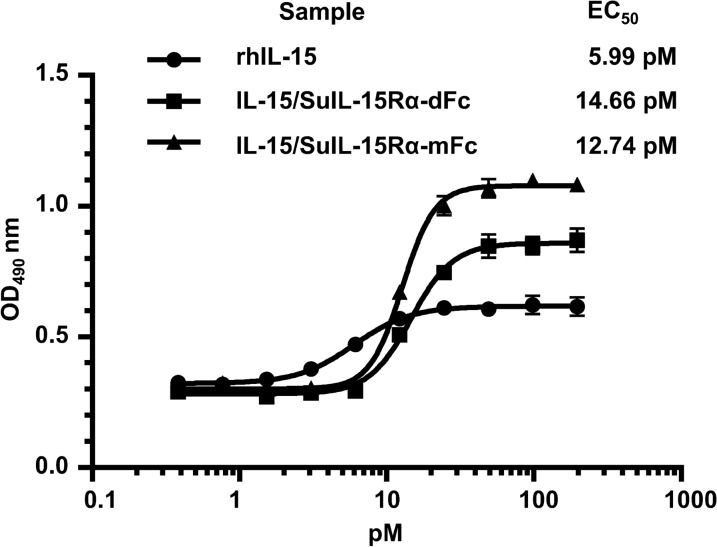
In vitro bioactivity of rhIL-15, IL-15/SuIL-15Rα-dFc and IL-15/SuIL-15Rα-mFc. A CTLL-2 cell proliferation assay was used to evaluate the biological activity of the rhIL-15 and IL-15/SuIL-15Rα-IgG4 Fc complexes. The EC_50_ values were calculated using the four-parameter nonlinear logistic regression model. All data points are the means ± standard deviation of triplicate OD values. The results are representative of at least three experiments

The in vitro biological activity of rhIL-15 and IL-15/SuIL-15Rα-IgG4 Fc complexes was evaluated by CTLL-2 cell proliferation assay. As shown in Fig. 3, the EC_50_ of rhIL-15 was 5.99 pM, and the EC_50_ values of IL-15/SuIL-15Rα-dFc and IL-15/SuIL-15Rα-mFc were 14.66 pM and 12.74 pM, respectively. The results demonstrate that the activity of IL-15/SuIL-15Rα-mFc is equivalent or even slightly better than that of IL-15/SuIL-15Rα-dFc for CTLL-2 cell proliferation.

### Prolonged half-lives of IL-15/SuIL-15Rα-IgG4 Fc complexes

**Fig. 4:**
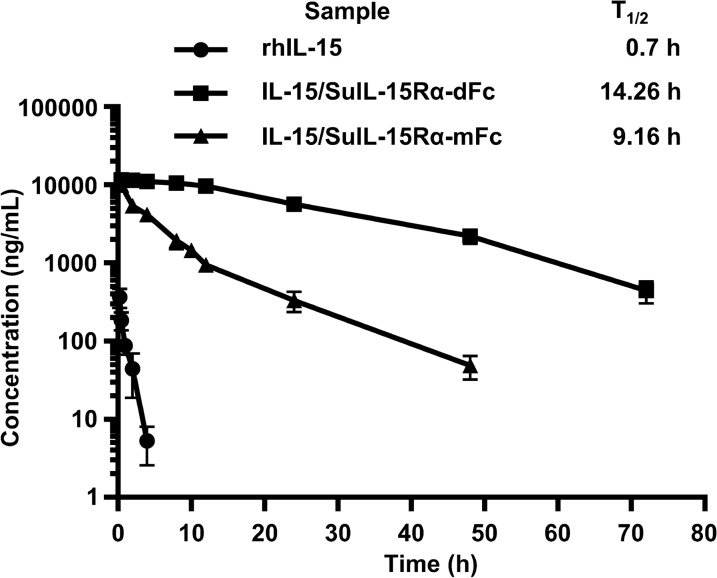
Pharmacokinetics analysis of rhIL-15, IL-15/SuIL-15Rα-dFc and IL-15/SuIL-15Rα-mFc. C57BL/6 J mice (male, 6–8 weeks of age, n = 4–9 for each group) were intravenously administered 0.28 mg/kg rhIL-15 or 1 mg/kg IL-15/SuIL-15Rα-IgG4 Fc complexes, and blood samples were collected at the indicated time points after injection. The concentration of IL-15 in serum was measured by ELISA. All data points are the means ± standard deviation of the concentrations

To evaluate the half-lives of IL-15/SuIL-15Rα-IgG4 Fc complexes, C57BL/6 J mice were intravenously injected with 0.28 mg/kg rhIL-15 or 1 mg/kg IL-15/SuIL-15Rα-IgG4 Fc complexes. Blood was collected at the indicated time points after injection, and the concentration of IL-15 in serum was measured by ELISA. The data were analyzed using the noncompartmental model with PKsolver software [28]. As shown in Fig. 4, the half-life of rhIL-15 was 0.7 h, while the half-lives of IL-15/SuIL-15Rα-dFc and IL-15/SuIL-15Rα-mFc were 14.26 h and 9.16 h, respectively. The results indicate that compared with rhIL-15, the half-lives of IL-15/SuIL-15Rα-IgG4 Fc complexes extend remarkably, and the plasma concentration of IL-15 decreases more slowly. Although the half-life of IL-15/SuIL-15Rα-mFc was shorter than that of IL-15/SuIL-15Rα-dFc, it was still much longer than that of rhIL-15.

### Effects of IL-15/SuIL-15Rα-IgG4 Fc complexes on the expansion of mouse lymphocytes

**Fig. 5:**
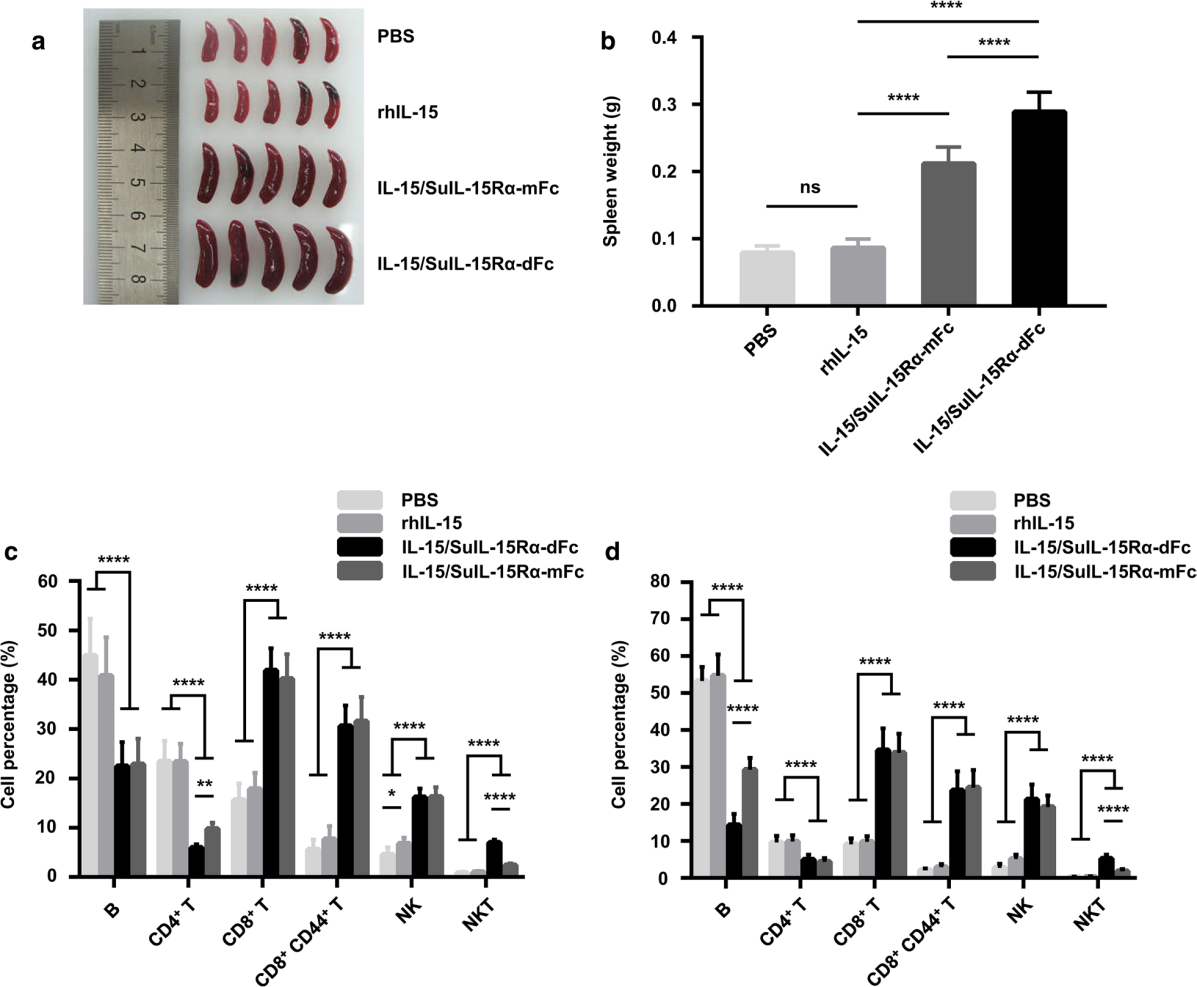
In vivo bioactivity of rhIL-15, IL-15/SuIL-15Rα-dFc and IL-15/SuIL-15Rα-mFc. C57BL/6 J mice (male, 6–8 weeks of age, n = 10–14 for each group) were intravenously injected with PBS, 0.28 mg/kg rhIL-15 or 1 mg/kg IL-15/SuIL-15Rα-IgG4 Fc complexes. Seventy-two hours after treatment, spleens and blood samples were collected and prepared for flow cytometry. **a** Representative photo of spleens separated from each group (n = 5). **b** Spleen weight statistics. **c** The percentage of each indicated cell subset in splenocytes. **d** The percentage of each indicated cell subset in peripheral blood cells. The data shown in b, c, and d are combined from three independent experiments, and all data points are the means ± standard deviation. ns, not significant; *: p < 0.05; **: p < 0.01; ***: p < 0.001 and ****: p < 0.0001

IL-15 is a potent immunostimulatory factor that is critical for the development, proliferation, and function of NK cells, NKT cells, and CD8^+^ T cells. To compare the bioactivity of the IL-15/SuIL-15Rα-IgG4 Fc complexes with that of rhIL-15 in vivo, C57BL/6 J mice received a single intravenous injection of 0.28 mg/kg rhIL-15 or 1 mg/kg IL-15/SuIL-15Rα-IgG4 Fc complexes. As shown in Fig. 5, compared to the slight immunostimulatory effect of rhIL-15, the treatment of the two IL-15/SuIL-15Rα-IgG4 Fc complexes led to the appearance of splenomegaly (Fig. 5a, b), and significantly elevated the percentages of NK cells, NKT cells, and CD8^+^ CD44^+^ T memory cells to a much higher level in spleens (Fig. 5c) and blood (Fig. 5d), in line with previous studies [10, 11]. Although the increased percentage of above immune cells by the two IL-15/SuIL-15Rα-IgG4 Fc complexes was similar, the spleens of IL-15/SuIL-15Rα-dFc-treated mice were larger than those of IL-15/SuIL-15Rα-mFc-treated mice, which may be due to the longer half-life of IL-15/SuIL-15Rα-dFc. In addition, the percentage of NKT cells was higher in the spleens and blood of IL-15/SuIL-15Rα-dFc-treated mice than in those of IL-15/SuIL-15Rα-mFc-treated mice, while the percentage of CD4^+^ T cells in the spleens and B cells in blood tended to be lower.

### Effects of IL-15/SuIL-15Rα-IgG4 Fc complexes on the activation and proliferation of NK cells and CD8^+^ T cells in human PBMCs

**Fig. 6:**
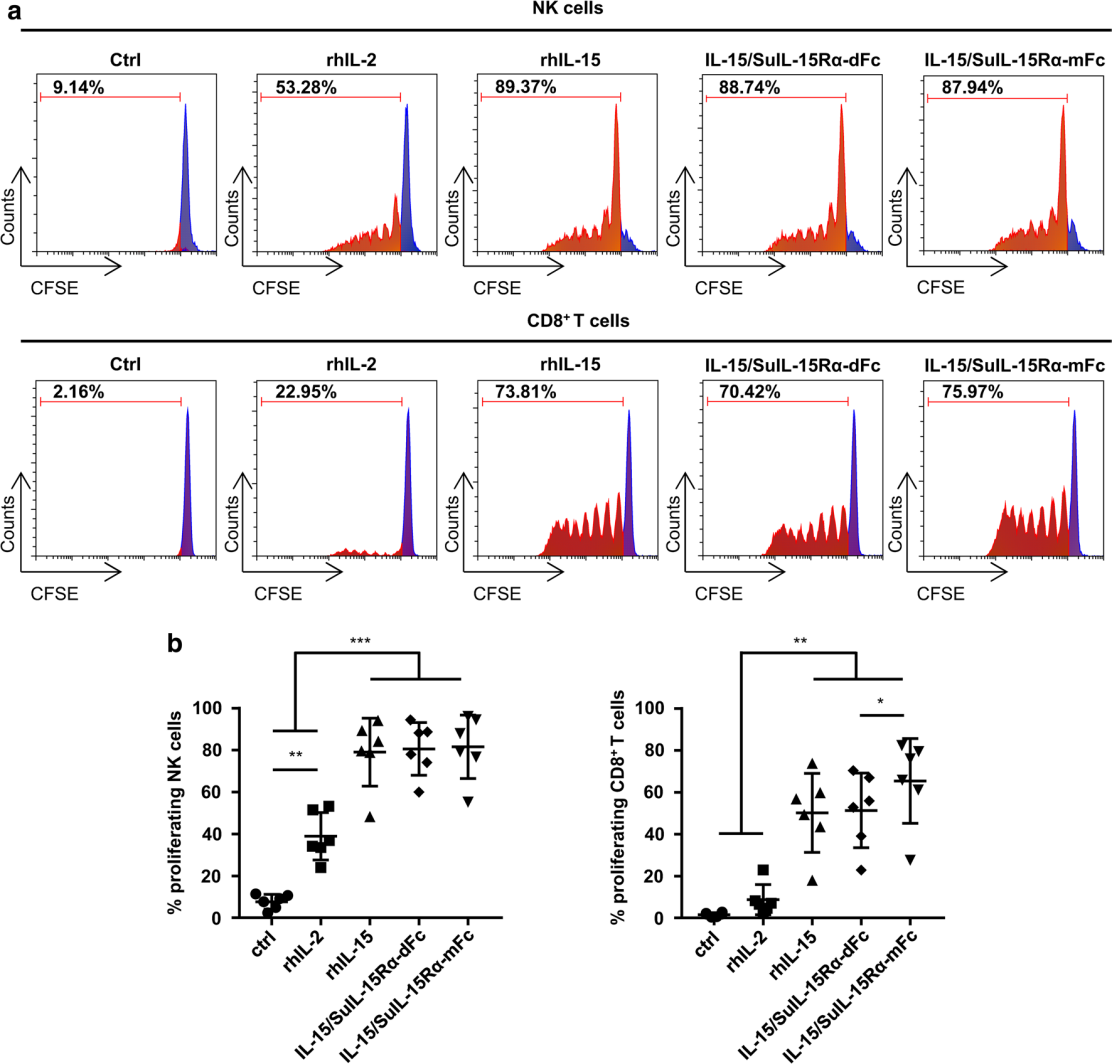
Effects of rhIL-2, rhIL-15, IL-15/SuIL-15Rα-dFc, and IL-15/SuIL-15Rα-mFc on NK and CD8^+^ T cell proliferation. Human PBMCs from healthy donors (n = 6) were cultured for 7 days in the absence or presence of 50 IU/mL rhIL-2, 10 ng/mL rhIL-15 or 35.7 ng/mL IL-15/SuIL-15Rα-IgG4 Fc complexes, and the proliferation of CFSE^+^ NK cells and CFSE^+^ CD8^+^ T cells was measured by flow cytometry. **a** Results from one representative healthy donor. **b** Statistics on the proliferation of NK cells or CD8^+^ T cells from all healthy donors. All data points are means ± standard deviation. *: p < 0.05; **: p < 0.01; ***: p < 0.001

To explore the effects of the IL-15/SuIL-15Rα-IgG4 Fc complexes on the activation and proliferation of human NK cells and CD8^+^ T cells, PBMCs from healthy donors were freshly isolated and treated with 50 IU/mL rhIL-2, 10 ng/mL rhIL-15 or 35.7 ng/mL IL-15/SuIL-15Rα-IgG4 Fc complexes for 24 h. PBS–treated cells served as control. As shown in Table. 2, for NK cells in PBMCs, rhIL-15 treatment significantly increased the expression levels of several molecules, such as activation receptors (CD69, NKG2D, NKp44, and 4-1BB), inhibitory receptors (Tim-3 and TIGIT), cytotoxicity-related molecules (Granzyme B, CD107a, and TRAIL), the pro-inflammatory cytokine IFN-γ and the proliferation marker Ki67, whereas rhIL-2-treated cells exhibited a slight increase in these markers. IL-15/SuIL-15Rα-dFc and IL-15/SuIL-15Rα-mFc treatment exhibited comparable immunostimulatory activities for NK cells compared to rhIL-15 treatment. Similar results were also observed for CD8^+^ T cells in PBMCs (Table. 3). The expression levels of IFN-γ and Ki67 of CD8^+^ T cells were increased to a similar degree after treatment with rhIL-15 or the two IL-15/SuIL-15Rα-IgG4 Fc complexes. To further confirm the proliferative activity of the two IL-15/SuIL-15Rα-IgG4 Fc complexes, CFSE-labeled PBMCs were incubated with the indicated proteins mentioned above for 7 days (Fig. 6a, b). NK cells and CD8^+^ T cells exhibited more potent proliferation in the groups treated with rhIL-15 and the two IL-15/SuIL-15Rα-IgG4 Fc complexes. In addition, the proliferative activity of IL-15/SuIL-15Rα-mFc was slightly higher compared with that of IL-15/SuIL-15Rα-dFc for CD8^+^ T cells. Overall, these results suggest that the two IL-15/SuIL-15Rα-IgG4 Fc complexes could significantly enhance the activation and proliferation of NK cells and CD8^+^ T cells.

**Table 2:** Effects of rhIL-2, rhIL-15 and IL-15/SuIL-15Rα-IgG4 Fc complexes on NK cell phenotypic markers

Marker	Ctrl	rhIL-2	rhIL-15	dFc	mFc
CD69	5.8 ± 3.0	28.3 ± 13.9	81.4 ± 18.6	85.4 ± 11.8	78.6 ± 22.0
CD25	2.9 ± 0.6	3.5 ± 0.5	24.0 ± 8.6	24.6 ± 7.8	22.7 ± 8.2
CD94	65.6 ± 29.0	73.4 ± 22.2	86.2 ± 10.6	84.8 ± 12.1	85.2 ± 11.6
NKG2D	75.6 ± 12.8	91.6 ± 5.4	96.1 ± 2.0	95.6 ± 2.7	95.7 ± 2.5
NKp44	2.3 ± 0.6	5.0 ± 1.5	9.2 ± 3.8	12.0 ± 3.6	8.9 ± 3.4
CD107a	6.4 ± 2.1	11.2 ± 6.4	20.3 ± 9.7	21.7 ± 12.0	21.2 ± 7.9
IFN-γ	1.4 ± 0.5	2.1 ± 0.7	10.0 ± 2.7	10.8 ± 2.1	11.7 ± 3.8
TRAIL	20.0 ± 14.8	34.0 ± 13.4	60.1 ± 17.1	62.7 ± 15.4	60.8 ± 18.1
4-1BB	2.3 ± 0.9	6.2 ± 3.6	15.3 ± 6.9	16.9 ± 7.8	15.0 ± 7.6
Granzyme B	89.7 ± 4.4	96.3 ± 1.6	98.8 ± 0.8	98.9 ± 0.8	98.8 ± 0.9
Ki67	4.9 ± 1.7	7.3 ± 2.5	16.2 ± 2.6	16.6 ± 2.8	16.0 ± 2.2
Tim-3	9.0 ± 4.8	33.5 ± 11.7	57.1 ± 13.7	58.4 ± 15.4	55.4 ± 15.7
TIGIT	29.3 ± 2.8	40.0 ± 5.9	55.4 ± 11.0	56.0 ± 7.9	55.7 ± 10.5

Human PBMCs from healthy donors (n = 5) were cultured for 24 h in the absence or presence of 50 IU/mL rhIL-2, 10 ng/mL rhIL-15 or 35.7 ng/mL IL-15/SuIL-15Rα-IgG4 Fc complexes, and the expression of selected markers on NK cells was analyzed by flow cytometry. The percentages of NK cells that expressed the corresponding markers are listed in the table. dFc: IL-15/SuIL-15Rα-dFc; mFc: IL-15/SuIL-15Rα-mFc

**Table 3:** Effects of rhIL-2, rhIL-15 and IL-15/SuIL-15Rα-IgG4 Fc complexes on CD8^+^ T cell phenotypic markers

Marker	Ctrl	rhIL-2	rhIL-15	dFc	mFc
IFN-γ	1.6 ± 0.3	1.7 ± 0.4	6.5 ± 2.3	8.6 ± 4.1	8.4 ± 3.1
Granzyme B	20.7 ± 7.5	22.9 ± 8.3	24.5 ± 7.4	25.3 ± 7.7	25.4 ± 8.0
Perforin	31.8 ± 7.3	37.5 ± 7.7	40.7 ± 10.4	40.6 ± 11.9	40.1 ± 11.8
Ki67	0.9 ± 0.4	1.5 ± 0.7	2.6 ± 0.9	3.2 ± 2.1	2.5 ± 0.9

Human PBMCs from healthy donors (n = 5) were cultured for 24 h in the absence or presence of 50 IU/mL rhIL-2, 10 ng/mL rhIL-15 or 35.7 ng/mL IL-15/SuIL-15Rα-IgG4 Fc complexes, and the expression of selected markers on CD8^+^ T cells was analyzed by flow cytometry. The percentages of CD8^+^ T cells that expressed the corresponding markers are listed in the table. dFc: IL-15/SuIL-15Rα-dFc; mFc: IL-15/SuIL-15Rα-mFc

## Discussion

In this study, we designed two IL-15 complexes. IL-15/SuIL-15Rα-dFc had a similar structural design to ALT-803 but with the substitution of IgG4 Fc for IgG1 Fc, and several mutations were introduced into the IL-15 and Fc fragments to remove the *N*-glycosylation and reduce effector functions through IgG4 Fc/FcγR interactions. In addition, we generated IL-15/SuIL-15Rα-mFc with a smaller size and simpler configuration using a linker. We successfully expressed the two complexes using the *P. pastoris* expression system, which has the industrial potential to produce functional proteins at a low cost. Although we obtained sufficient proteins for this research, the protein yield could be further improved by optimizing the process parameters, including the methanol feed rate, induction temperature and pH [29].

After a multistep purification process, we obtained the two IL-15/SuIL-15Rα-IgG4 Fc complexes with high purity. SDS-PAGE and Western blot analysis revealed the IL-15/SuIL-15Rα-mFc preparation exhibited relatively higher purity, which suggested that the monomeric form may be easier to obtain uniform products than the dimeric form in production. To the best of our knowledge, this is the first report on the successful expression and purification of functional IL-15 superagonists using *P. pastoris*.

It has been reported that compared with the IL-15 monomer, the half-lives of various IL-15 complexes are significantly prolonged. For example, IL-15·sIL-15Rα had a half-life of 4 h (i.p.) [30], ALT-803 had a half-life of 7.5 h (i.v.) or 7.7 h (s.c.) [31], and IL-15·sIL-15Rα/Fc had a half-life of 13.1 h (i.p.) [12]. In this study, a pharmacokinetic assessment in C57BL/6 J mice (i.v.) showed that the half-lives of the two IL-15/SuIL-15Rα-IgG4 Fc complexes (14.26 h for IL-15/SuIL-15Rα-dFc and 9.16 h for IL-15/SuIL-15Rα-mFc) were significantly extended compared with that of rhIL-15 (0.7 h). The half-life of rhIL-15 was consistent with previous reports (30–40 min) [11, 12], while the variation in the half-life values of IL-15 complexes mentioned above may be due to various routes of administration and analysis methods used in different studies. The half-life of IL-15/SuIL-15Rα-mFc was approximately half that of IL-15/SuIL-15Rα-dFc, which may be due to restrictive kidney filtration; specifically, proteins less than 70 kDa are more likely eliminated by kidney filtration [32].

IL-15 is a pivotal cytokine that preferentially stimulates the proliferation, activation, and cytolytic activity of NK cells and CD8^+^ T cells. Previous studies suggested that the N72D mutation and the association of IL-15 and IL-15Rα could enhance the biological activity of IL-15. However, the CTLL-2 proliferation assay showed that rhIL-15 activity was superior to that of the two IL-15/SuIL-15Rα-IgG4 Fc complexes. Since CTLL-2 cells express the mouse IL-15Rα, it is reasonable to speculate that this observation might be caused by the fact that the complex of rhIL-15/mouse IL-15Rα has higher affinity to mouse IL-15Rβ/γ_c_ complex compared to human IL-15/SuIL-15Rα-IgG4 Fc complexes. Besides, N72D effect is restricted to human rather than mouse IL-15Rβ and may masked by the presence of the high affinity IL-15Rα subunit, which is consistent with a previous report [7]. Moreover, the activity of IL-15/SuIL-15Rα-mFc was slightly higher than that of IL-15/SuIL-15Rα-dFc. This finding is likely due to the relief of steric hindrance between the two “arms” of dimeric Fc [25], which made the binding of IL-15/SuIL-15Rα-mFc to IL-15Rβγ_c_ more flexible. In contrast, murine in vivo studies demonstrated that compared with the rhIL-15 treatment group, treatment with the two IL-15/SuIL-15Rα-IgG4 Fc complexes significantly promoted proliferation of NK cells and memory CD8^+^ T cells in the spleen and blood, in line with previous studies [11]. The enhanced in vivo activity observed in the two IL-15/SuIL-15Rα-IgG4 Fc complex treatment groups benefits from the increased activity by the association between IL-15 and the IL-15Rα-sushi domain and the prolonged half-lives in circulation. Interestingly, we also observed that NKT cell proliferation in the IL-15/SuIL-15Rα-dFc treatment group was more obvious than that in the IL-15/SuIL-15Rα-mFc treatment group in both the spleens and blood of mice. We speculated that this result might be related to the dimer structure of IL-15/SuIL-15Rα-dFc. More work needs to be done to help clarify the precise mechanism. Furthermore, we also observed changes in the expression levels of phenotypic markers in NK cells and CD8^+^ T cells using PBMCs from healthy donors after treatment with rhIL-15 and the two IL-15/SuIL-15Rα-IgG4 Fc complexes. Consistent with previous studies [33], several activating receptors on NK cells, including CD69, NKG2D, NKp44, and 4-1BB, and functional molecules, such as IFN-γ, Granzyme B, CD107a, and TRAIL, were increased after treatment, which expression were decreased in cancer patients as a sign of NK cell exhaustion [34, 35]. Elevated expression levels were also observed in the inhibitory receptors Tim-3 and TIGIT. Although Tim-3 has been considered an exhaustion-associated marker, other studies have implicated Tim-3 as a mature marker on NK cells [36] that is associated with NK cell activation and function [37, 38]. In addition, rhIL-15 and the two IL-15/SuIL-15Rα-IgG4 Fc complex treatments also upregulated the expression of the proliferation marker Ki67, which was consistent with the results of the CFSE proliferation assay. Interestingly, we observed that most NK cells proliferated in response to the stimulation by rhIL-15 or the two IL-15/SuIL-15Rα-IgG4 Fc complexes. However, a majority of these cells were stagnated in the first generation of division, while the remaining cells continued to divide in a limited fashion. This finding is likely due to the heterogeneity of human samples coming from different donors, given that one of our samples showed continuous cell division in most NK cells. In addition, CD8^+^ T cells also exhibited moderate improvement in activation and proliferation after treatment with rhIL-15 or the two IL-15/SuIL-15Rα-IgG4 Fc complexes. Moreover, IL-15/SuIL-15Rα-mFc promoted CD8^+^ T cell proliferation more significantly than rhIL-15 and IL-15/SuIL-15Rα-dFc.

## Conclusions

In this study, we designed and successfully expressed two IL-15/SuIL-15Rα-IgG4 Fc complexes in *P. pastoris* with full biological activities and extended half-lives, which may serve as immunotherapy agents for clinical development. Our study also suggests that, given that IL-15/SuIL-15Rα-mFc exhibits potent bioactivity, prolonged half-life, and high purity available in production, it could be an alternative form of IL-15 superagonists expressed in *P. pastoris* for further research and applications.

## Methods

### Strains, plasmids, and antibodies

The *Pichia pastoris* strain GS115, *Escherichia coli* strain DH5α, and expression vectors pPIC9 and pPICZα were purchased from Invitrogen (Carlsbad, CA). The template plasmids pPIC9-SuIL-15Rα and pPICZα-IgG4-Fc (with mutations S228P, L235E, and N297Q) were constructed and preserved in our laboratory. Anti-IL-15 was purchased from Boster Bio (Pleasanton, CA, PB0249). HRP-conjugated antibody to rabbit IgG was purchased from Biolegend (San Diego, CA, 406401). Mouse anti-human IgG4-HRP conjugate was purchased from Abcam (Cambridge, MA, ab99817). In addition, rhIL-15 was purchased from PeproTech (Rocky Hill, NJ, 200-15), and rhIL-2 was purchased from Jiangsu Kingsley Pharmaceutical Co., Ltd. (Jiangsu, China). The IL-15 capture antibody (MAB647) and IL-15 detection antibody (BAM247) used in ELISA were purchased from R&D Systems (Minneapolis, MN).

### Mice and cell lines

C57BL/6 J mice were purchased from the Model Animal Research Center of Nanjing University and were housed under specific-pathogen free conditions at the University of Science and Technology of China. All animal experiments were approved by the Ethics Committee of the University of Science and Technology of China. The CTLL-2 cell line was a gift from Prof. Zhigang Tian. CTLL-2 cells were cultured in RPMI medium modified (HyClone, Logan, UT) supplemented with 10% FBS (Biological Industries, Beit HaEmek, Israel), 200 IU/mL rhIL-2, 100 U/mL penicillin and 100 µg/mL streptomycin (Sangon Biotech, Shanghai, China) at 37 °C in a 5% CO_2_ incubator.

### Construction of expression vectors

The human IL-15 gene was amplified from PBMCs of healthy donors. Site-directed mutagenesis by overlap extension PCR was used to introduce four mutations (N71Q, N72D, N79Q, and N112Q) into the coding region of IL-15. The coding sequence of mutated IL-15 was inserted into the zeocin-resistant vector pPICZα to construct the expression vector pPICZα-IL-15.

The fusion gene SuIL-15Rα-dFc was constructed by linking the sushi domain of human IL-15Rα and the human IgG4 Fc fragment through the hinge region of IgG4. Briefly, Fw-suIL15Rα-xho I and Rv-suIL15Rα-hinge primers were used to amplify fragment 1 from the template plasmid pPIC9-SuIL-15Rα, and Fw-suIL15Rα-hinge and Rv-IgG4 Fc-not I primers were used to amplify fragment 2 from the template plasmid pPICZα-IgG4-Fc, which already had the three mutations mentioned above. Fw-suIL15Rα-xho I and Rv-IgG4 Fc-not I primers were used to splice the two fragments to obtain the fusion gene SuIL-15Rα-dFc. The fusion gene SuIL-15Rα-mFc was constructed by linking the sushi domain of human IL-15Rα and the human IgG4 Fc fragment via a linker (Gly_4_Ser)_3_. Briefly, Fw-suIL15Rα-xho I and Rv-suIL15Rα-linker-IgG4 Fc primers were used to amplify fragment 3 from the template plasmid pPIC9-SuIL-15Rα, and Fw-SuIL15Rα-linker-IgG4 Fc and Rv-IgG4 Fc-not I primers were used to amplify fragment 4 from the template plasmid pPICZα-IgG4-Fc. Fw-suIL15Rα-xho I and Rv-IgG4 Fc-not I primers were used to splice the two fragments to obtain the fusion gene SuIL-15Rα-mFc. The primers used in this study were listed in Additional file 1: Table S1.

The two fusion genes and the ampicillin-resistant vector pPIC9 were digested by the restriction enzymes *Xho* I and *Not* I, ligated by DNA ligase enzyme to construct the expression vectors, and named pPIC9-SuIL-15Rα-dFc and pPIC9-SuIL-15Rα-mFc, respectively.

### Expression of the IL-15/SuIL-15Rα-IgG4 Fc complexes in *P. pastoris*

The expression vector pPICZα-IL-15 was linearized by the restriction enzyme *Sac* I, while pPIC9-SuIL-15Rα-dFc and pPIC9-SuIL-15Rα-mFc were linearized by the restriction enzyme *Sal* I. To coexpress the mutated IL-15 with SuIL-15Rα-dFc or with SuIL-15Rα-mFc in the same strain, the linearized vector pPIC9-SuIL-15Rα-dFc or pPIC9-SuIL-15Rα-mFc was transformed into *P. pastoris* stain GS115, and then the cell suspension was spread on histidine-deficient minimal dextrose (MD) plates. These positive clones were screened by Western blotting, and the highest expression clone was subjected to further transformation with linearized pPICZα-IL-15. The transformants were plated onto YPD plates with 1 mg/mL zeocin (Sigma). The positive clones were screened by dot blotting using an anti-human IL-15 antibody, and the high-expression clones were further selected by Western blotting using an anti-human IgG4-HRP conjugate. One of these high-expression clones was used in pilot-scale fermentation.

### Fermentation and purification

Fed-batch fermentation was performed according to Invitrogen’s Pichia Fermentation Process Guidelines using a 14-L NBS BioFlo fermenter (Eppendorf, Hamburg, Germany), in line with a previous report [39]. Specifically, 400-mL *P. pastoris* seed culture was inoculated into 6-L BMGY medium containing 4% (w/v) glycerol. The culture temperature was set to 28 °C, and the pH was maintained at 6.0 by adding ammonium hydroxide. Once all the glycerol was consumed, 50% (w/v) glycerol feed containing 12 mL/L PTM1 solution was continuously added into the fermenter until the cellular yield reached 180–220 g/L,then glycerol feed was terminated and proceeded into the methanol fed-batch phase. According to the standard three-step protocol recommended by Invitrogen, methanol induction was started by adding 100% (w/v) methanol containing 12 mL/L PTM1 solution and set the methanol feed rate to 3.6 mL/hour/L(initial fermentation volume). When the culture was fully adapted to methanol utilization (about 2–4 h), the methanol feed rate was then increased to 7.3 mL/hour/L(initial fermentation volume). Two hours later, the methanol feed rate was further increased to 10.9 mL/h/L(initial fermentation volume) and maintained throughout the remainder of the fermentation. The culture temperature was set to 25 °C, the stirring rate was controlled at 1000 rpm, and the DO was maintained at approximately 25–30%. At the end of fermentation, the pH of the fermentation broth was adjusted to 7.5 with ammonium hydroxide, and the supernatant was collected by centrifugation at 10,000×*g* for 20 min. The supernatant was then filtered through 0.45-μm and 500-kDa microfiltration cartridges using the FlexStand system (GE Healthcare Life Sciences, Chicago, IL). A HiTrap MabSelect affinity chromatography column was used to capture the target proteins from the supernatant, and a HiLoad 26/600 Superdex 200-pg size exclusion chromatography column was used to further purify proteins using the AKTA Pure25 System (GE Healthcare Life Sciences). Endotoxin levels in the final products were evaluated using the Tachypiens Amebocyte Lysate (TAL) purchased from Bioendo (Fujian, China) according to manufacturer’s instructions. The purified protein complexes were then filtered through 0.22-μm filters and stored at −80 °C.

### Characterization of the purified protein complexes

The purified protein samples were separated by 12% SDS-PAGE under reducing conditions, and the gel was stained with Coomassie brilliant blue. For immunoblot analysis, protein samples were separated by 12% SDS-PAGE under reducing conditions, transferred onto a PVDF membrane, and probed with the IL-15 primary antibody followed by anti-rabbit HRP-conjugated secondary antibody to detect IL-15 or probed with the mouse-anti-human IgG4-HRP conjugate to detect IgG4 Fc. The protein bands were visualized using a chemiluminescence detection kit (Advansta, Menlo Park, CA) according to the manufacturer’s instructions.

### CTLL-2 cell proliferation assay

A CTLL-2 cell proliferation assay was used to determine the biological activity of the rhIL-15 and IL-15/SuIL-15Rα-IgG4 Fc complexes. In brief, CTLL-2 cells in logarithmic growth phase were washed thrice with RPMI medium containing 10% FBS (basal medium) and then seeded into 96-well culture plates at 1 × 10^4^ cells per well (60 μL). The rhIL-15 and IL-15/SuIL-15Rα-IgG4 Fc complexes were serially diluted with basal medium and added into the plates at 40 μL per well. After 48 h of incubation, the cell viability was measured using the MTT colorimetric assay. The results were analyzed by GraphPad Prism software, and EC_50_ values were calculated using the four-parameter fit logistic equation.

### Pharmacokinetic evaluation

C57BL/6 J mice (male, 6–8 weeks of age) were randomly and equally divided into three groups. Each group of mice was intravenously injected with 0.28 mg/kg rhIL-15 or 1 mg/kg IL-15/SuIL-15Rα-IgG4 Fc complexes (a molar equivalent dose of rhIL-15). Blood samples were collected at the indicated time points after injection: 0.25, 0.5, 1, 2, and 4 h for the group treated with rhIL-15; 0.5, 2, 4, 8, 12, 24, 48, and 72 h for the group treated with IL-15/SuIL-15Rα-dFc; and 0.5, 2, 4, 8, 10, 12, 24, and 48 h for the group treated with IL-15/SuIL-15Rα-mFc. Plasma IL-15 levels were detected by ELISA using the IL-15 capture antibody and IL-15 detection antibody. The serum half-lives of rhIL-15 and IL-15/SuIL-15Rα-IgG4 Fc complexes were analyzed using PKsolver software in a noncompartmental model [28].

### In vivo lymphocyte proliferation assay

C57BL/6 J mice (male, 6–8 weeks of age) were randomly divided into four groups. Each experimental group of mice was intravenously injected with 0.28 mg/kg rhIL-15 or 1 mg/kg IL-15/SuIL-15Rα-IgG4 Fc complexes. The PBS group was used as a negative control. After 72 h, blood and spleens were collected from each group to obtain a single cell suspension. The cells were washed with PBS and resuspended in PBS containing 10% rat serum (Future, Guangzhou, China) at 4 °C for 30 min and then stained with the appropriate antibodies at 4 °C for 30 min in the dark. After staining, the cells were washed thrice with PBS and then detected by the CytoFLEX Flow Cytometer (Beckman Coulter). The data were analyzed using CytExpert software. The antibodies used in this study were listed in Additional file 1: Table S2.

### Human PBMC isolation and in vitro culture

Human peripheral blood mononuclear cells (PBMCs) were isolated from the peripheral blood of healthy donors with informed consent at the Blood Centre of Anhui Province using Ficoll-Hypaque (Solarbio, Beijing, China) density gradient centrifugation. For analysis of NK cell and CD8^+^ T cell activation, PBMCs were cultured in RPMI medium supplemented with 10% FBS in the absence or presence of 50 IU/mL rhIL-2, 10 ng/mL rhIL-15 or 35.7 ng/mL IL-15/SuIL-15Rα-IgG4 Fc complexes (a molar equivalent dose of rhIL-15) for 24 h. For analysis of NK cell and CD8^+^ T cell proliferation, PBMCs were labeled with 5 μM CFSE (Biolegend) and then incubated for 7 days in the indicated conditions mentioned above. After incubation, cells were harvested, washed with PBS, and then resuspended in PBS containing 10% mouse serum (Future, Guangzhou, China) at 4 °C for 30 min prior to staining with fluorescently conjugated antibodies at 4 °C for 30 min in the dark. For intracellular staining, cells were incubated with 10 ng/mL monensin (Abcam) for 4 h at 37 °C in a 5% CO_2_ incubator followed by staining for extracellular markers. Cells were then fixed, permeabilized (Invitrogen) and stained for intracellular molecules. The stained cells were subsequently washed, detected by a CytoFLEX Flow Cytometer and analyzed by CytExpert software. The antibodies used in this study were listed in Additional file 1: Table S2.

### Statistical analysis

Statistical analysis was performed in GraphPad Prism 6.0 software. One-way analysis of variance (ANOVA) was used to determine statistical significance in our study. Data are presented as the means ± standard deviation. ns: not significant; *: p < 0.05; **: p < 0.01; ***: p < 0.001; ****: p < 0.0001.

## Supplementary Information

**Additional file 1: Table S1.** List of primers used for fusion genes SuIL-15Rα-dFc and SuIL-15Rα-mFc construction. **Table S2.** List of antibodies used for flow cytometry.

## Data Availability

The datasets used and/or analysed during the current study are included in this article and its supplementary information files, and are available from the corresponding author on reasonable request.

## Abbreviations

IL: Interleukin
NK: Natural killer
IL-15Rα: IL-15 receptor α
Su: Sushi domain
IL-15Rβ: IL-15 receptor β
*P. pastoris*: *Pichia pastoris*
rh: Recombinant human
PBMCs: Peripheral blood mononuclear cells
FcγR: Fcγ receptor
AOX 1: Alcohol oxidase 1
HIS4: Histidinol dehydrogenase
WCW: Wet cell weight
MW: Molecular weight
PBS: Phosphate buffered saline
FBS: Fetal bovine serum
