# Supplementary material for: Development of IL-15/IL-15Rα sushi domain-IgG4 Fc complexes in Pichia pastoris with potent activities and prolonged half-lives
Source: Microb Cell Fact. 2021 Jun 9;20:115. doi: 10.1186/s12934-021-01605-3 (PMC8190845; doi:10.1186/s12934-021-01605-3)
Supplement: Supplementary file 1 — Additional file 1: Table S1. List of primers used for fusion genes SuIL-15Rα-dFc and SuIL-15Rα-mFc construction. Table S2. List of antibodies used for flow cytometry. [file 12934_2021_1605_MOESM1_ESM.docx]

**Supplementary Table 1. List of primers used for** **fusion genes SuIL-15Rα-dFc and SuIL-15Rα-mFc construction.**

| **Primer name** | **Sequence** |
| --- | --- |
| Fw-suIL15Rα-xho I | GGGTATCTCTCGAGAAAAGAATCACGTGCCCTCCCCCCAT |
| Rv-suIL15Rα-hinge | GACCATATTTGGACTCGTCTCTAATGCATTTG |
| Fw-suIL15Rα-hinge | CAAATGCATTAGAGACGAGTCCAAATATGGTC |
| Rv-IgG4 Fc-not I | TATGCGGCCGCCTATTTACCCAGAGACAGGGAG |
| Rv-suIL15Rα-linker-IgG4 Fc | GTCCCCCTTCGAACTCAGGTGCAGAACCACCACCACCAGAACCACCACCACCAGAACCACCACCACCGTCTCTAATGCATTTGAGACTG |
| Fw-SuIL15Rα-linker-IgG4 Fc | CAGTCTCAAATGCATTAGAGACGGTGGTGGTGGTTCTGGTGGTGGTGGTTCTGGTGGTGGTGGTTCTGCACCTGAGTTCGAAGGGGGAC |

**Supplementary Table 2. List of antibodies used for flow cytometry.**

| **Antibodies** | **Source** | **Cat#** |
| --- | --- | --- |
| FITC anti-mouse CD3ε | Biolegend | 100305 |
| PerCP/Cy5.5 anti-mouse CD8a | Biolegend | 100734 |
| PE/Cy7 anti-mouse CD4 | Biolegend | 100528 |
| APC anti-mouse/human CD44 | Biolegend | 103012 |
| Brilliant violet 785^TM^ anti-mouse NK1.1 | Biolegend | 108749 |
| Alexa Fluor® 700 anti-mouse CD19 | Biolegend | 115528 |
| APC/Cy7 anti-mouse CD45 | Biolegend | 103116 |
| FITC anti-human CD4 | Biolegend | 300506 |
| PE anti-human CD8 | Biolegend | 344706 |
| PerCP/Cy5.5 anti-human CD3 | Biolegend | 300430 |
| Brilliant violet 785^TM^ anti-human CD56 | Biolegend | 362550 |
| APC/Cy7 anti-human CD45 | Biolegend | 368516 |
| PE anti-human TRAIL | Biolegend | 308206 |
| PE/Cy7 anti-human CD25 | Biolegend | 356108 |
| APC anti-human CD69 | Biolegend | 310910 |
| FITC anti-human Tim-3 | Biolegend | 345022 |
| PE/Cy7 anti-human 4-1BB | Biolegend | 309818 |
| Alexa Fluor® 647 anti-human CD107a | Biolegend | 328612 |
| FITC anti-human CD94 | Biolegend | 305504 |
| PE/Cy7 anti-human NKp44 | Biolegend | 325116 |
| APC anti-human TIGIT | Biolegend | 372706 |
| APC anti-human NKG2D | Biolegend | 320808 |
| PE/Cy7 anti-human IFNγ | Biolegend | 506518 |
| Alexa Fluor® 647 anti-human/mouse Granzyme B | Biolegend | 515406 |
| PE/Cy7 anti-human Perforin | Biolegend | 353316 |
| APC anti-human Ki67 | Biolegend | 350514 |
| DAPI (4',6-Diamidino-2-Phenylindole, Dilactate) | Biolegend | 422801 |
